# The Use of Genome-Wide eQTL Associations in Lymphoblastoid Cell Lines to Identify Novel Genetic Pathways Involved in Complex Traits

**DOI:** 10.1371/journal.pone.0022070

**Published:** 2011-07-15

**Authors:** Josine L. Min, Jennifer M. Taylor, J. Brent Richards, Tim Watts, Fredrik H. Pettersson, John Broxholme, Kourosh R. Ahmadi, Gabriela L. Surdulescu, Ernesto Lowy, Christian Gieger, Chris Newton-Cheh, Markus Perola, Nicole Soranzo, Ida Surakka, Cecilia M. Lindgren, Jiannis Ragoussis, Andrew P. Morris, Lon R. Cardon, Tim D. Spector, Krina T. Zondervan

**Affiliations:** 1 Genetic and Genomic Epidemiology Unit, The Wellcome Trust Centre for Human Genetics, University of Oxford, Oxford, United Kingdom; 2 Bioinformatics Core, The Wellcome Trust Centre for Human Genetics, University of Oxford, Oxford, United Kingdom; 3 Departments of Medicine and Human Genetics, McGill University, Montréal, Canada; 4 Twin Research and Genetic Epidemiology Unit, St Thomas' Hospital Campus, King's College London, London, United Kingdom; 5 Genomics Laboratory, The Wellcome Trust Centre for Human Genetics, University of Oxford, Oxford, United Kingdom; 6 Institute of Epidemiology, Helmholtz Zentrum München, German Research Center for Environmental Health, München, Germany; 7 Broad Institute of Harvard and MIT, Massachusetts General Hospital, Harvard Medical School, Boston, Massachusetts, United States of America; 8 Institute for Molecular Medicine Finland FIMM, University of Helsinki and National Institute for Health Welfare, Helsinki, Finland; 9 Human Genetics, Wellcome Trust Sanger Institute, Hinxton, Cambridge, United Kingdom; 10 GlaxoSmithKline, King of Prussia, Pennsylvania, United States of America; University of Texas M. D. Anderson Cancer Center, United States of America

## Abstract

The integrated analysis of genotypic and expression data for association with complex traits could identify novel genetic pathways involved in complex traits. We profiled 19,573 expression probes in Epstein-Barr virus-transformed lymphoblastoid cell lines (LCLs) from 299 twins and correlated these with 44 quantitative traits (QTs). For 939 expressed probes correlating with more than one QT, we investigated the presence of eQTL associations in three datasets of 57 CEU HapMap founders and 86 unrelated twins. Genome-wide association analysis of these probes with 2.2 m SNPs revealed 131 potential eQTLs (1,989 eQTL SNPs) overlapping between the HapMap datasets, five of which were in *cis* (58 eQTL SNPs). We then tested 535 SNPs tagging the eQTL SNPs, for association with the relevant QT in 2,905 twins. We identified nine potential SNP-QT associations (P<0.01) but none significantly replicated in five large consortia of 1,097–16,129 subjects. We also failed to replicate previous reported eQTL associations with body mass index, plasma low-density lipoprotein cholesterol, high-density lipoprotein cholesterol and triglycerides levels derived from lymphocytes, adipose and liver tissue. Our results and additional power calculations suggest that proponents may have been overoptimistic in the power of LCLs in eQTL approaches to elucidate regulatory genetic effects on complex traits using the small datasets generated to date. Nevertheless, larger tissue-specific expression data sets relevant to specific traits are becoming available, and should enable the adoption of similar integrated analyses in the near future.

## Introduction

The availability of high throughput and low cost genotyping technologies have lead to recent successes of genome-wide association (GWA) studies in mapping genes contributing to various complex traits including diabetes, lipids and bone mineral density (BMD) and obesity [1]–[3]. Many consistently replicated associations between clinical phenotypes and genetic variants have been found to date. However, most of these studies – particularly those involving quantitative traits (QTs) - require very large sample sizes to detect modest effects which explaining only a small fraction of the heritability associated with these traits; furthermore, they do not provide experimental data supporting the functional and regulatory consequences of the associations [4]. Linkage disequilibrium (LD) across the associated region and time-consuming experiments to gain functional evidence make identification of the causal variants difficult. A common approach employed by various studies to gain insight into the possible regulatory role of replicated disease-associated Single Nucleotide Polymorphisms (SNPs) is to investigate their correlation with transcript levels. For instance, Moffatt et al. (2007) found that the most significant SNPs associated with childhood asthma risk in a large LD region with 19 candidate genes accounted for 29.5% of the variance of *ORMDL3* transcript levels measured in lymphoblastoid cell lines (LCLs) and thus *ORMDL3* was prioritized as a primary biological candidate for the asthma locus [5]. In a similar approach, a SNP near *TNFRSF11B*, strongly associated with BMD was found to be associated with a 50% decrease in expression levels of *TNFRSF11B* in LCLs, providing a putative mechanism for the SNP-trait association [2].

These approaches still rely, however, on the detection of replicated SNP-disease associations in the first instance. Several recent studies of the genetic basis of regulatory variation (expression Quantitative Trait Loci (eQTL) studies) have shown that *cis*-acting genetic variants can be strongly associated with gene expression levels generated from various human samples including LCLs [6]–[11], whole blood [12], fresh lymphocytes [13], abdominal fat [12] and liver tissue [14], and some of these *cis*-acting variants were reported to be correlated with a few clinical QTs [2], [5], [12]–[16]. The general question remains, however, to what extent gene expression traits that are likely to be under stronger genetic influence than ‘downstream’ complex traits [13], [17], can help in uncovering novel genetic pathways involved in phenotypic variation. An additional question is whether LCLs, used extensively to date in eQTL discovery studies [6]–[11] are suited to elucidating clinically relevant expression patterns.

In humans, LCLs have been commonly used in eQTL studies [6]–[11], mainly because they are an easily accessible source of a single cell type, in which immediate environmental influences and variation manifested by other cell types on expression are minimized compared to *ex vivo* samples thus decreasing noise and allowing - in theory - a more powerful investigation of genetic influences. However, LCLs -being removed from immediate environmental influences such as inflammatory responses –are transformed and cultured under artificial conditions and may not represent the natural gene expression state *in vivo*. In addition, the large percentage of pauciclonality in LCLs combined with widespread monoallelic expression has been shown to lead to highly differential expression profiles between LCLs and *ex vivo* cells [18], [19]. These differences between cell lines and natural tissues might significantly reduce effect sizes of both QT/disease-gene expression and eQTL associations. As long as LCLs mirror the relevant patterns of regulation, an integrated analysis of replicated eQTLs with clinical phenotypes could provide a more powerful and informative approach to elucidate the genetic regulation of complex traits.

Such integrated analyses - ideally involving evidence from multiple datasets - are, however, methodologically not straightforward. Potential problems include the unknown relevance of LCL expression profiles for the phenotype in question. Indeed comprehensive collections of more relevant human tissues are not commonly available for many clinical phenotypes. Moreover, several studies have highlighted cell type- or tissue specific genetic effects, where others found that a substantial numbers of eQTLs are shared across tissues [12], [20]. A few studies compared the overlap of eQTLs found in LCLs and primary tissues and found that a large number of eQTLs detected in LCLs can also be detected in primary tissues [21]–[23].

In addition, differences in probe annotation and SNP tag coverage across datasets may hamper comparisons across studies. Gene expression profiles obtained from different platforms in different laboratories analysed with the same annotation and statistical methods [24]–[26] may improve the reproducibility of eQTL studies [19], [25], [27].

In this study, we investigated the potential of combining gene expression data from LCLs and genotype data to uncover associations with a more regulatory and functional role between clinical traits and underlying genetic variants. Specifically, rather than focussing solely on the direct association between genetic variants and a single clinical trait, or between genetic variants and expression phenotypes, we sought to determine whether an integrated approach would enable the detection of clinically relevant associations. For this purpose, we used clinical, LCL expression, and genotyping data from the UK Adult Twin registry, a longitudinal epidemiological study of 11,000 twins (mostly female) for whom extensive clinical, anthropometric, life-style, and demographic information, as well as a wide range of biological measurements have been collected [28]. In addition, we aimed to detect robust eQTLs by using the twin data as well as three sources of publicly available HapMap LCL expression data generated on Illumina platforms but re-analysed using identical methodology since they used different array versions and were produced in different laboratories. Although the size of the datasets employed for eQTL detection were only likely to enable the detection of *cis* eQTLs relevant to QTs - the focus of our study - we also present relevant results for *trans* effects. Our study is the first to investigate the utility of eQTLs in a large number of QTs (N = 44), which include BMD, anthropometric, metabolic and fat-related traits.

## Results

**Figure 1:**
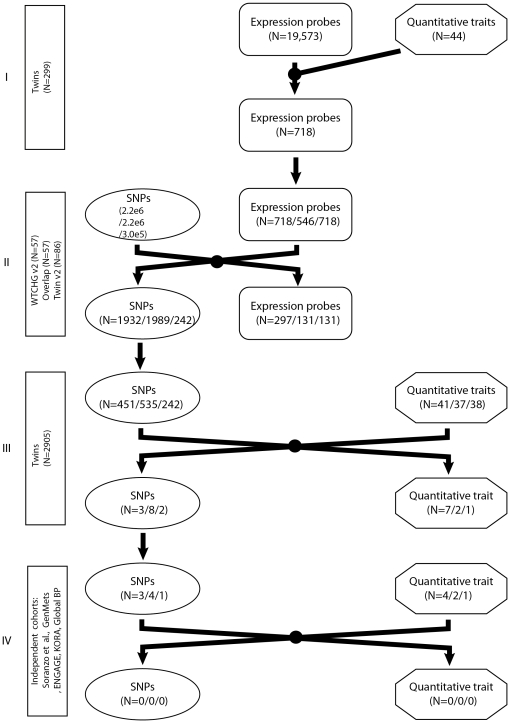
The study design comprises four different analysing stages. Stage I: Correlation analysis between 44 QTs and 19,573 detected gene expression levels in 299 female twins profiled in LCLs. Stage II: eQTL detection of nominally significant QT-correlated probes in 57 CEU HapMap individuals and 86 unrelated twins. Stage III: confirmation of QT-SNP association in 2,905 female twins. Stage IV: Replication of QT-SNP association in large, independent cohorts.

The study design comprised four analysis stages (Figure 1): I) prioritisation of clinically relevant expression probes through correlation of 44 QTs with gene expression in LCLs of 299 female twins; II) identification of SNPs associated with QT-correlated expression probes (eQTLs) in 57 CEU HapMap individuals and 86 unrelated twins; III) confirmation of QT-SNP association in the larger cohort of 2,905 female twins; IV) replication of QT-SNP association in large, independent cohorts. The hypothesis behind this design was that focussing eQTL SNP detection on expression traits correlated with QTs should enrich for QT-SNP associations. This screening step could increase power of detection not only because expression traits are likely to be under stronger genetic control than more downstream QTs, but also because it potentially reduces the extent of multiple testing otherwise inherent to genome-wide QT association approaches.

**Figure 2:**
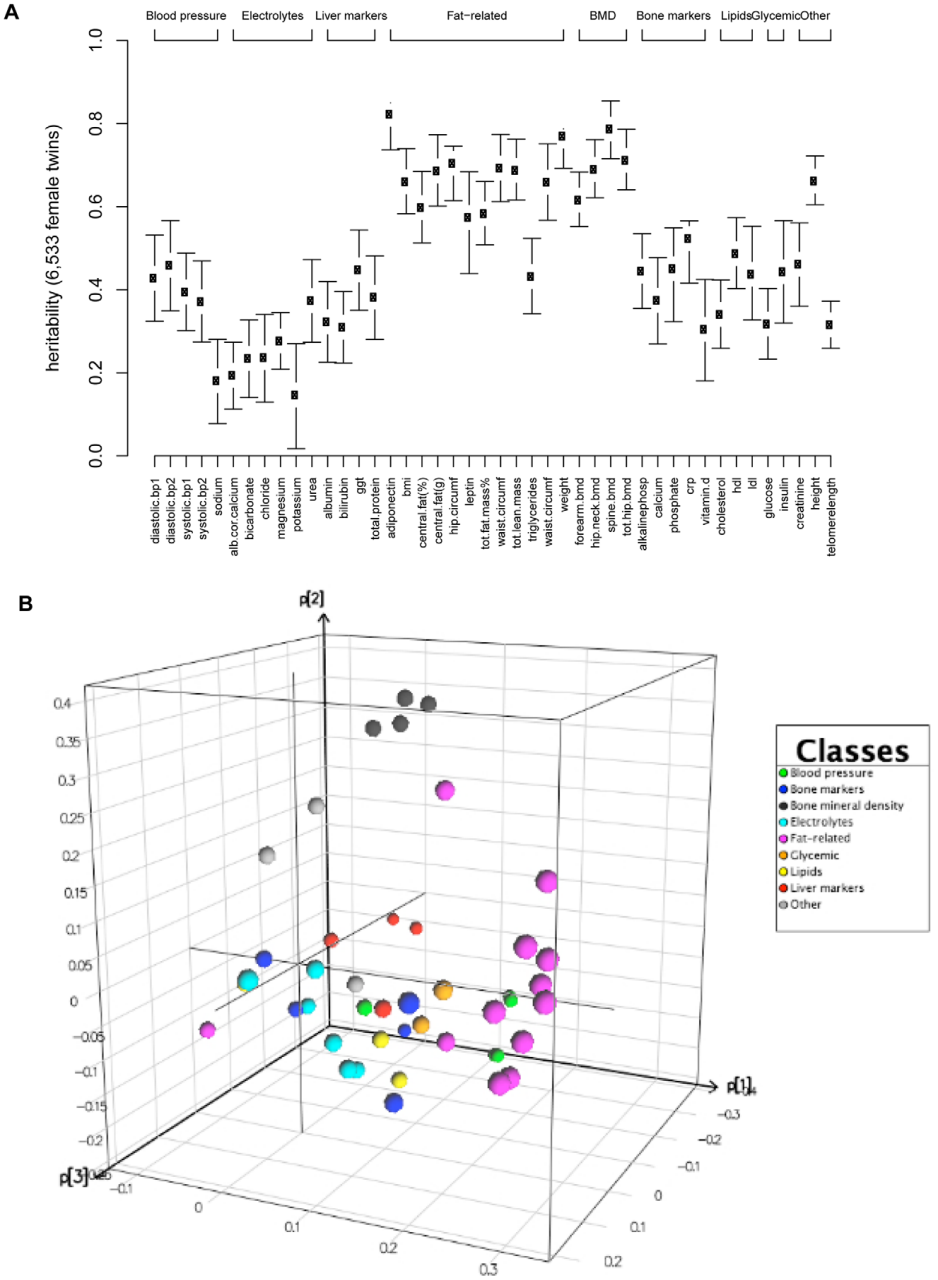
Heritability estimates and PCA of QTs. A) Heritability estimates with 95% confidence intervals of 44 QTs ordered by clinical subgroups. Abbreviations are: bp1 = first blood pressure measurement; bp2 = second blood pressure measurement; alb cor calcium = albumin corrected calcium; ggt = gamma-glutamyl transpeptidase; bmi = body mass index; circumf = circumference; tot.fat.mass% = total fat mass (%); bmd = bone mineral density; alkalinephosph = alkalinephosphatase; crp = C-reactive protein; hdl = high-density protein cholesterol; ldl = low-density protein cholesterol B) PCA of 44 QTs. Three clusters of QTs were discriminated: fat related QTs including weight, BMI, total fat mass (g), total fat mass (%), central fat (g), central fat (%), hip circumference, waist circumference and leptin, BMD QTs including forearm, hip, femoral neck and spine BMD and a cluster with four blood pressure QTs.

We selected 44 QTs measured in the twin cohort including anthropometric traits, BMD, fat-related traits, electrolytes, liver function markers, bone markers, lipids and glycemic traits (Methods, Text S1). We first estimated the heritabilities of these QTs in the entire twin cohort of 6,533 female twins (Figure 2A). In general, the lowest heritabilities were found for electrolyte measurements (h^2^: 0.15–0.37) and highest heritabilities were found for fat-related (h^2^: 0.43–0.82) and BMD traits (h^2^: 0.62–0.79). To investigate the correlation structure of the 44 QTs, principal components analysis (PCA) on the 44 QTs was performed discriminating three clusters: 9/12 fat-related traits, four BMD traits and four blood pressure traits (Figure 2B, Figure S1).

### Stage I: Correlations between QTs and gene expression levels in female twins

In Stage I, we generated gene expression profiles for a subset of 299 female Caucasian twins with available LCLs using Illumina Human WG-6 V2 Sentrix BeadArrays containing 46,713 probes. A total of 19,573 (42%) expression probes (11,854 Ensembl genes) were detected in at least 10% of the individuals (Figure 1) and had no SNPs in their sequence. We tested these expression probes for association with each of the QTs using linear mixed models, adjusting for the confounding effect of RNA Integrity Number, cell culture duration, age, and for correlation of measurements due to experimental design (see Methods). Given its potential confounding effect, we investigated the effect of smoking on gene expression measurements, but found that only one expression probe-QT correlation was mediated by smoking (Text S1, Table S1). In total, 939 expression probes (4.8%) correlated with at least one QT at a nominal significance value of p<10^−3^, and 137 (0.7%) at p<10^−4^ (Table 1). Of the 939 expression probes, 160 correlated with 2–8 QTs at p<10^−3^, giving a total of 1,176 expression probe-QTs correlations (Table S2). The five expression probes correlated with ≥5 QTs were correlated with blood pressure and fat-related traits. The 939 expression probes nominally correlated with QTs at p<10^−3^ were taken to Stage II. Assuming independence between the QTs, the estimated false discovery rate (FDR) was 0.73 across the 19,573 probes and 44 QTs. Given the correlations between many of the QTs, this estimate was likely to be a substantial overestimate. At this stage, however, we did not adjust for multiple testing because: i) results of Stage I were taken forward to the subsequent confirmation Stages II–IV during which appropriate correction was made; and ii) substantial correlations exist between QTs (Figure S1) and between expression probes and such adjustments would be extremely stringent.

**Table 1:** Number of correlations between 44 QTs and 19,573 expression probes (11,854 Ensembl genes) at different significance levels for 299 female twins.

*P* value threshold	No. of QTs (N = 44)	No. of correlated probes (N = 19,573)	No Ensembl genes	No. of probe-QT correlations
<10^−3^	44	939	703	1,176
<10^−4^	34	137	91	161
<10^−5^	12	15	12	18
<10^−6^	3	3	3	3

### Stage II: eQTL analysis in the HapMap and Twin datasets

To uncover consistent eQTL signals underlying expression signals correlated with QTs, we analysed and annotated three LCL expression datasets from 57 CEU HapMap founders (Wellcome Trust Centre for Human Genetics (WTCHG) version 1 (V1), WTCHG version 2 (V2), Sanger V1 [11]) that were measured on two Illumina Beadchips (V1 and V2) in two laboratories using the same methods (Text S1, Table S3 and S4). In addition, we conducted eQTL detection in 86 unrelated twins from Stage I (Twin V2) for which we had genotypes and LCL expression data available. High reproducibility among the same HapMap individuals was observed for 184 identical probes that were detected in at least 10% of individuals in both WTCHG V1 and WTCHG V2 datasets (spearman ρ = 0.96 (standard deviation = 0.015)). Although our main focus - given the size of the datasets - was on detecting *cis* eQTLs, we also included *trans* signals for the purpose of contrast and completeness. Associations in *cis* were defined as SNP-probe associations where SNPs were located within a region 1 Mb upstream or downstream of the expression probe midpoint. We considered all other associations as associations in *trans* [11]. The rationale for the 1 Mb window was that most of the eQTLs lie either within genes or close to the gene [7], [17], [29].

For the eQTL analyses, we selected 718 of the 939 expression probes (640 Ensembl genes) from Stage I that were detected in at least 10% of the HapMap individuals (WTCHG V2) and had well-mapped autosomal locations on NCBI build 36 (Figure 1). Our primary aim was to leverage the combined evidence of eQTL detection across the different datasets, with overlap in signals between SNP-expression probe pairs from the HapMap datasets providing evidence of technical replication, and overlap between SNP-expression probe pairs from HapMap and the twin dataset providing evidence of biological replication. However, overlap analyses could involve only those 546/718 (76%) of the V2 probes (594 Ensembl genes) which had the same Ensembl transcript target as at least one probe on the Illumina V1 Beadchip.

We tested the 546 probes (corresponding to 1,050 V1 expression probes) for eQTL association in the three HapMap datasets using a nominal significance threshold of p<10^−3^ in all three datasets (‘overlap’ analysis). To capture the increased coverage of RefSeq genes on the V2 Beadchips compared to V1 Beadchips, we also conducted eQTL analyses using the WTCHG V2 and Twin V2 datasets only for all prioritised 718 V2 probes. For the WTCHG V2 and Twin V2 analyses, we applied a nominal significance threshold of p<5^*^10^−7^, a threshold applied in GWA studies of a single trait [3]. We fitted additive models between normalised expression values of each of the selected expression probes and 2.2 million SNPs downloaded from www.HapMap.org [30] in the HapMap datasets and 296,308 SNPs in Twin V2 (see Methods). We tested additive models only as a previous study showed that dominance had a minimal effect on gene transcription [7].

After removal of eQTLs with different directional effects, the overlap analysis yielded 131 expression probes (135 Ensembl genes) associated with at least one SNP at p<10^−3^ in all three HapMap datasets (1,989 eQTL SNPs). Five of the expression probes (five Ensembl genes) provided 58 *cis* eQTL associations (Table 2). The remaining 129 expression probes (133 Ensembl genes) resulted in 1,959 *trans* eQTL associations. In the WTCHG V2 dataset, we identified 297 probes (251 Ensembl genes) with a total of 1,954 eQTL associations (p<5^*^10^−7^, 1,932 eQTL SNPs); five of the 297 probes (four Ensembl genes) had 39 eQTL associations in *cis* (Table 2).

**Table 2:** Number of probes with eQTL SNPs derived from GWA analysis of 546 (overlap analysis) and 718 (WTCHG V2) expression probes in 57 HapMap founders and 86 unrelated twins.

Dataset	eQTL p value threshold	No. mapped probes with eQTL^a^	No. Ensembl genes	No. eQTLs	No SNPs	No. probes with *cis* eQTL SNPs	No. *cis* eQTLs^b^
***Overlap eQTL analysis***							
WTCHG V2/WTCHG V1/Sanger V1	<10^−3^	131	135	2,017	1,989	5	58
***Dataset-specific eQTL analysis***							
WTCHG V2	<5*10^−7^	297	251	1,954	1,932	5	39
Twin V2	<5*10^−7^	131	110	252	242	10	42
***Meta-eQTL analysis***							
WTCHG V2+Twin V2	<5*10^−7^	85	73	102	101	9	18

a Probes annotated to genomic location in build 36.

b *cis* eQTL were defined as an SNP-probe association where the distance from probe genomic midpoint to SNP genomic location was less than or equal to 1 Mb; all other eQTLs were defined as *trans* eQTLs.

In the Twin V2 dataset, 131 probes (110 Ensembl genes) with 252 eQTL associations (242 eQTL SNPs) were found; ten of the 131 probes (10 Ensembl genes) provided 42 eQTL associations in *cis*. The corresponding FDR for *cis* eQTL analysis of 718 probes in the WTCHG V2 analysis and Twin V2 at a pvalue of 5*10^−7^ was 0.01 and 0.001, respectively.

Only four eQTL associations from the overlap analysis (two probes, two Ensembl genes) and 229 eQTL associations (164 probes, 142 Ensembl genes) from the WTCHG V2 were replicated in the Twin V2 dataset at the 1*10^−3^ threshold. Fifteen of the 164 probes (13 Ensembl genes) provided 39 eQTL associations in *cis*. Combining the WTCHG V2 and Twin V2 datasets, we tested these 229 eQTL associations in a fixed-effects meta-analysis [31]. After checking for heterogeneity, these analyses resulted in 102 eQTL associations (85 probes, 73 Ensembl genes) at the 5*10^−7^ threshold of which nine probes (seven Ensembl genes) showed 18 eQTL associations in *cis*.

To investigate whether the reduced sets of expression probes resulting from Stages I and II are more likely to be functionally related to the QTs, we summarised them in “Biological Process” Gene Ontology (GO) categories using DAVID [32] and assessed heritability distributions (see Methods). The 546 expression probes from Stage I showed an enrichment of common GO terms such as “cell fate specification” (p = 4.5*10^−4^, FDR P = 0.24) whereas the 131 probes identified from the overlap analysis (Stage II) and the 85 probes identified from the meta-analysis showed an excess of the GO term ‘cellular lipid metabolism’ (GO:00044255, p = 2.0*10^−3^, FDR P = 0.11) and ‘negative regulation of response to stimulus’(GO:0048585, p = 4.0*10^−4^, FDR P = 0.10) compared to the 18,838 detected probes, respectively.

**Figure 3:**
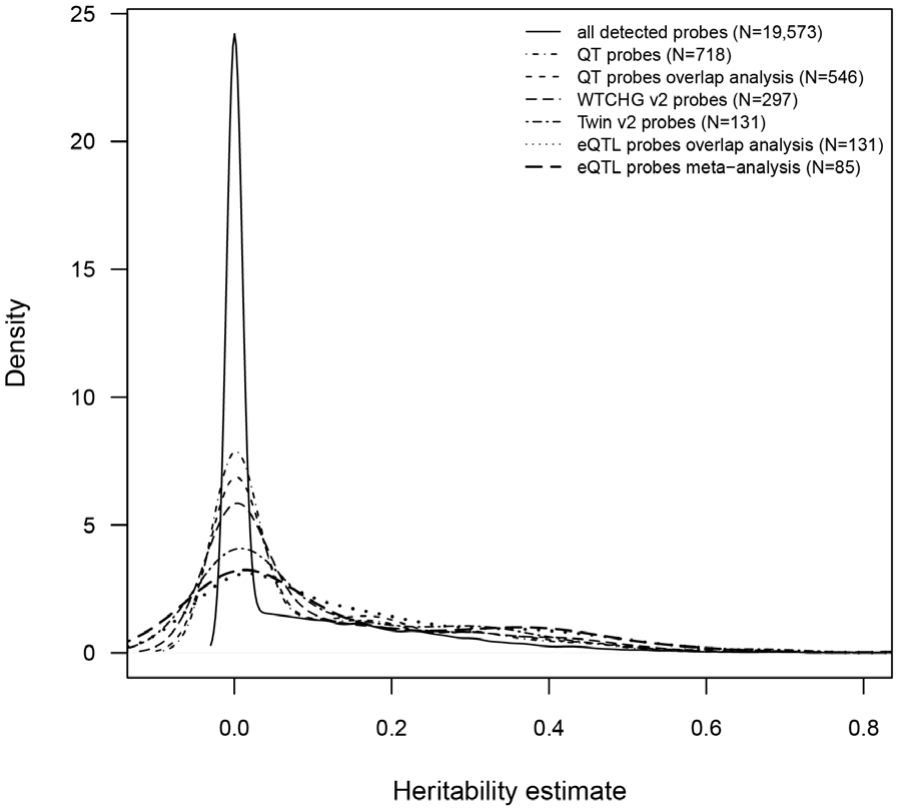
Density plot of heritability estimates of gene expression probes for different analysis stages.

Despite the small sample size, the frequency of expression probes with heritabilities >0.2 increased throughout the selection strategy, from 15% of all 19,573 detected probes, 20% of the 718 QT-correlated probes, 21% of the 546 the QT correlated probes annotated to WTCHG V1 probes, 23% of the 297 eQTL probes identified from WTCHG V2 dataset, 27% of the 131 eQTL probes from Twin V2 dataset, to 31% of 131 eQTL probes from the overlap analysis and 31% of 85 eQTL probes from the meta-analysis (Figure 3). The increasing percentage of heritable probes and the enrichment of GO terms suggested that our filtering strategy up to Stage II was successful in increasing the proportion of true-positive relative to false-positive QT-related signals.

### Stage III: Confirmation of associations between eQTL SNPs and QTs in twin cohort

In Stage III, nominally significant eQTL SNPs and those in LD (r^2^>0.8) were tested for association with the QTs with which the relevant expression probes were previously correlated (Stage I), in the larger cohort of 2,905 female twins (Figure 1). For these twins whole genome genotyping was available from the twin cohort employing 296,308 SNPs with the Illumina HumanHap 300 k Duo Beadchip (Illumina, San Diego, USA) after appropriate quality checks (see Methods). eQTL and GWA signals were considered overlapping for SNPs on the GWA chip that were in LD with the eQTL SNP. For the overlap analysis, 535 SNPs on the GWA chip were in LD (r^2^>0.8) with 1,482/1,989 eQTL SNPs (75%) from Stage II (see Methods). In the WTCHG V2 analysis, 451 SNPs were in LD (r^2^>0.8) with 1,357/1,932 (70%) eQTL SNPs (Table S5). The percentage of tagged eQTL SNPs in the overlap analysis was greater than for the WTCHG V2 analysis, possibly explained by the increased percentage of rarer eQTL SNPs in the WTCHG V2 analysis: 54% of untagged SNPs had a minor allele frequency (MAF) ≤0.1 compared to 33% in the overlap analysis (Figure S2).

We examined the association between 535 SNPs from the overlap analysis with 37 QTs (corresponding to 141 probe-QT correlations and 1,498 eQTL associations), 451 SNPs from WTCHG V2 analysis with 41 QTs (268 probe-QT correlations and 1,370 eQTL associations), 242 SNPs with 38 QTs from Twin V2 (161 probe-QT correlations and 252 eQTL associations) and 101 SNPs with 36 QTs from the meta-analysis (105 probe-QT correlations and 102 eQTL associations). After removal of associations with inaccurate annotations or outliers in gene expression, we found 15 non-overlapping loci associated with 10 different QTs with a p-value<0.01 (Table 3). For these 16 SNP-QT associations with p<0.01, eQTL associations were tested in the full set of 86 twin families (161 individuals) to obtain a more precise estimate of significance (Table 3). For 8/15 of the associations, the constituting eQTL evidence was based on the overlap analysis (+/− Twin V2), whereas 5/15 associations had eQTL evidence from WTCHG V2 or Twin V2 only. Three associations showed eQTL evidence from the meta-analysis (one association was overlapping with the overlap analysis).

**Table 3:** Summary of results for QT-eQTL SNP associations reaching p<10^−2^ in Stage III, which were prioritised for independent replication in Stage IV.

	Stage I			Stage II					Stage III			Stage IV	
	QT- probe correlations twins (N = 299)			eQTL analysis					QT-eQTL SNP associations twin cohort (N = 2,905)			QT-eQTL SNP replication analysis	
QT	Expression probe	Probe target	p value	eQTL SNP	p values HapMap (N = 57)	p value twins (N = 161)	p value HapMap + twins	c*is*/*trans* eQTL	SNPs on GWA array	p value	FDR-p value	Cohort (N)	p value
***Overlap eQTL analysis (WTCHG V2, WTCHG V1, Sanger V1)***													
Phosphate	ILMN_27140	*CCDC58* (chr3)	5.4*10^−4^	rs10511409 (chr3)^e^	3.7*10^−4^, 2.3*10^−4^, 9.4*10^−4^	8.5*10^−4^		*cis*	rs10511409	7.7*10^−3^	0.50	KORA (1,756)	0.39
Chloride	ILMN_19002	*DPYD* (chr1)	4.7*10^−4^	rs17596670 (chr2)^f^	6.1*10^−4^, 1.2*10^−4^, 5.3*10^−4^	0.01		*trans*	rs313289	4.0*10^−3^	0.36	NA	
Height	ILMN_552	*DYRK3* (chr1)	2.3*10^−4^	rs716618 (chr6)	7.1*10^−4^, 5.5*10^−4^, 1.5*10^−4^	0.04, 0.05, 0.04,		*trans*	rs1482455, rs9342097, rs9353441	3.0*10^−3^, 4.4*10^−3^, 1.7*10^−3^	0.36, 0.36, 0.36	R'dam,1958BC, EPcohort, EPcases (5,828)	0.01, 0.02, 0.05
Crp	ILMN_18109	*RBPMS2* (chr15)	1.9*10^−5^	rs2652485 (chr5)	5.0*10^−4^, 6.7*10^−4^, 9.9*10^−4^	0.13		*trans*	rs2561858	2.6*10^−3^	0.36	Genmets (1,097)	0.45
Chloride	ILMN_23096	*SCGB3A1* (chr5)	7.8*10^−5^	rs13056641 (chr22)	1.9*10^−4^, 1.2*10^−4^, 7.8*10^−4^	0.29		*trans*	rs13056641	8.7*10^−3^	0.51	NA	
Triglycerides	ILMN_6736	*SH2D2A* (chr1)	2.7*10^−4^	rs2266917 (chr2)	4.1*10^−4^, 5.7*10^−4^, 4.3*10^−4^	0.40		*trans*	rs2266917	2.7*10^−3^	0.36	ENGAGE (16,129)	0.28
Hip circumference	ILMN_2829	*OAS1* (chr12)	3.0*10^−4^	rs3811876 (chr5)	1.8*10^−4^, 4.9*10^−4^, 3.5*10^−4^	0.95		*trans*	rs3811876	3.5*10^−3^	0.36	NA	
Potassium	ILMN_6528	*RAB31* (chr18)	4.7*10^−4^	rs1548691 (chr7)^a^	4.5*10^−4^, 6.0*10^−4^, 3.2*10^−6^	0.83		*trans*	rs2237496	7.8*10^−3^	0.50	NA	
***WTCHG V2 eQTL analysis***													
Diastolic blood pressure	ILMN_7391	*NPAS3* (chr14)	3.7*10^−4^	rs573179 (chr6)^b^	4.6*10^−7^	0.26, 0.65		*trans*	rs200969, rs149900	5.7*10^−5^, 3.4*10^−4^	0.03, 0.07	Global BP (26,644)	0.91, 0.26
Crp	ILMN_13910	*AMBP* (chr9)	2.9*10^−4^	rs9634941 (chr13)	1.2*10^−7^	0.74, 0.74		*trans*	rs966715, rs503283	7.9*10^−3^, 7.2*10^−3^	0.16, 0.16	Genmets (1,097)	0.38, 0.19,
Crp	ILMN_16204	*SMPDL3A* (chr6)	5.1*10^−4^	rs2079571 (chr17)^c^	6.6*10^−8^	0.22, 0.22		*trans*	rs4791449, rs1468482	3.1*10^−3^, 3.1*10^−3^	0.12, 0.12	Genmets (1,097)	0.50, 0.50
***Twin V2 eQTL analysis***													
Diastolic blood pressure	ILMN_19059	*KIAA1913* (chr6)	5.3*10^−4^	rs12150466 (chr17)	Not sign	8.5*10^−10^		*trans*	rs12150466	7.7*10^−4^	0.13	Global BP (26,644)	0.33
Femoral neck BMD	ILMN_15771	*YDJC* (chr 22)	3.5*10^−5^	rs7301360 (chr12)	Not sign	2.7*10^−5^		*trans*	rs7301360	1.5*10^−3^	0.16	NA	
Hip BMD	ILMN_15771	*YDJC* (chr 22)	1.7*10^−5^	rs7301360 (chr12)	Not sign	2.7*10^−5^		*trans*	rs7301360	5.8*10^−3^	0.50	NA	
***WTCHG V2 + Twin V2 eQTL analysis***													
Phosphate	ILMN_27140	*CCDC58* (chr3)	5.4*10^−4^	rs7647266 (chr3)^d^^,^ e	2.1*10^−4^	2.4*10^−5^	6.7*10^−8^	*cis*	rs7647266	1.4*10^−3^	0.11	KORA (1,756)	0.09
Chloride	ILMN_19002	*DPYD* (chr1)	4.7*10^−4^	rs313289 (chr2)^f^	6.6*10^−4^	0.01	3.4*10^−7^	*trans*	rs313289	4.0*10^−3^	0.17	NA	
Hip BMD	ILMN_15771	*YDJC* (chr 22)	1.7*10^−5^	rs10487243 (chr7)	5.8*10^−4^	0.05	2.4*10^−7^	*trans*	rs10487243	8.7*10^−3^	0.24	NA	

a rs1548691, rs2237496, rs3801416, rs4436006, rs6953784, rs6958841, rs6959221, rs7786661and rs7805746 are proxies for rs2237496 with r^2^ = 0.97.

b rs200978, rs200502, rs9468201 and rs9380062 are eQTL SNPs at this locus associated with diastolic blood pressure in the WTCHG V2 analysis (data not shown).

c rs10775390 (r^2^ = 1) and rs9910453 (r^2^ = 0.93) are proxies for rs2079571.

d rs6788377 and rs6810306 are proxies for rs7647266.

e rs10511409 and rs7647266 have moderate LD (r^2^ = 0.25).

f rs17596670 and rs313289 are in high LD (r^2^ = 0.89).

Only one of the QTs, phosphate, appeared to have contributing *cis* eQTL associations, for rs10511409 and rs7647266 (r^2^ = 0.25) on expression probe ILMN_27140, with supporting evidence from all HapMap datasets and the Twin V2 dataset or the meta-analysis, respectively. However, although ILMN_27140 had been selected because of its nominal correlation with phosphate levels in the 299 twins, the association between these SNPs and phosphate levels in 2,905 twins was non-significant after FDR correction (p>0.11).

All other QT-associated eQTL results were in *trans*, with marginal evidence for rs17596670 or rs313289 (r^2^ = 0.89) and ILMN_19002 (chloride concentration), and rs716618 and ILMN_552 (height). However, none of the corresponding QT-SNP associations were significant in the larger cohort of 2,905 twins after FDR correction [33]. Only the *trans* association from WTCHG V2 for diastolic blood pressure remained significant (FDR adjusted p = 0.03).

### Stage IV: Follow-up of SNP-QT associations in independent cohorts

We were able to test 9/16 QT-SNP associations (five QTs) for replication in five large-scale consortia datasets (Figure 1; Table 3): three GWAs from Soranzo *et al.* (2009) (height, N = 5,828); Genmets (C-reactive protein (crp), N = 1,097) [34]; ENGAGE (triglycerides, N = 16,129) [35]; GlobalBP (diastolic blood pressure, N = 26,644) [36]; and KORA (phosphate, N = 1,756) [37]. We were unable to identify suitably large replication cohorts for hip circumference, serum chloride and potassium, hip and femoral neck BMD. None of the cohorts had gene expression data available to replicate the gene-expression-QT correlation.

The *cis* associations between rs10511409 and rs7647266 and ILMN_27140 had been selected because of its nominal correlation with phosphate levels in the 299 twins. Subsequent analyses in an independent dataset of 1,756 individuals (KORA) did not provide evidence that these SNPs were associated with phosphate levels (p>0.09). Of the associations between QTs and SNPs which showed consistent *trans* eQTL evidence (for diastolic blood pressure, triglycerides, crp and height), only one association, between height and rs1482455 (tagging eQTL SNP rs716618 with r^2^ = 0.84) reached borderline significance (p = 0.01) in a meta-analysis of 5,828 females. However, this result was not significant after applying a Bonferroni correction allowing for the nine independent tests conducted (p<0.006).

### Investigation of published eQTL-QT associations

Several significant *cis* eQTL-QT associations have been published for QTs also included in our study, including associations with body mass index (BMI), plasma low-density lipoprotein cholesterol (LDL), high-density lipoprotein cholesterol (HDL) and triglycerides (Table S6) [12]–[14], [38]. Although none of these findings were derived from LCLs (fresh lymphocytes, liver or adipose tissue were used), we set out to investigate whether the associations could be detected in our datasets. Of the relevant 29 expression probes (25 genes) only 19 were expressed in the LCL dataset of 299 twins. None of the QT-expression probe correlations in the LCLs from the 299 female twins were nominally significant (p<10^−3^). Suggestive evidence was found for the correlations between the expression probes targeting *FADS1* and *FADS3* and HDL (p = 4*10^−3^) but eQTL analysis in the three HapMap or Twin V2 datasets did not confirm the reported significant *cis* eQTL associations.

### Power and sample size issues and potential limitations

**Figure 4:**
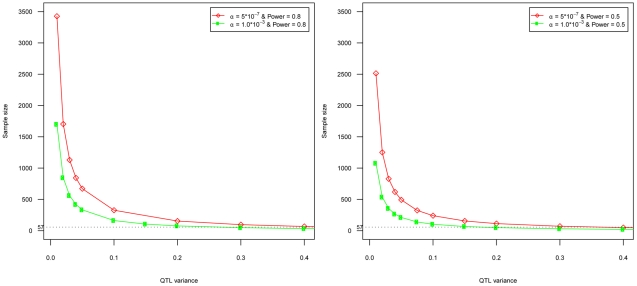
Sample sizes required to identify *cis* eQTL associations with 80% and 50% power at p<5^*^10^−7^ and p<1^*^10^−3^.

In any association study, power and sample size of the study design need careful consideration prior to analysis. Given the multi-stage design in our study, the complex correlation structure between QTs and between expression probes, the unknown effect sizes of QT-expression trait correlations (Stage I) and eQTLs (Stage II), the tagging of eQTL SNPs (Stage III) and the availability of suitable replication cohorts (Stage IV), simulation-based power calculations were unfeasible. Instead - given that an important aim of the study was to examine the utility of LCL-derived eQTL signals in relation to complex trait analysis - we adopted a much simplified scenario and calculated the likely power and sample sizes needed for *cis* eQTL detection. We estimated sample sizes required for a single expression trait in a GWA using single-SNP type I error rates of 5*10^−7^ and 1.0*10^−3^ with 80% and 50% power (Figure 4) [39].

Our results suggest that thousands of individuals would be needed to reliably detect effect sizes that explain a small proportion of the QTL variance (in additive models) of the expression trait at 5*10^−7^ or even 1.0*10^−3^ significance thresholds with >80% power (Figure 4). For the *cis* eQTLs found in Stage II, a large portion of the QTL variance appeared to be explained by the association ranging between 18%–51% in the overlap analysis, 37%–59% in the WTCHG V2 analysis and 27%–77% in the Twin V2 analysis.

## Discussion

Many GWAs have been conducted to find genetic variants underlying complex traits. A number of eQTL studies have identified genetic variants underlying gene expression mostly in LCLs. A few studies in humans have adopted an integrated approach using datasets in which genotypes and gene expression profiles – from adipose and liver tissue or lymphocytes – were correlated with a clinical trait [12]–[14]. Here, we investigated the potential of integrating gene expression data from LCLs and genotype data for a large number of clinical QTs (N = 44). Our multistage study, which incorporated eQTL signals from three sources of publicly available LCL expression data from 57 HapMap founders as well as an independent dataset of 161 twins, did not result in the identification of robust eQTL associations relevant to the 44 QTs. Using LCLs, we also failed to replicate previous reported eQTL associations with BMI, LDL, HDL and triglycerides levels derived from fresh lymphocytes, adipose and liver tissue [12]–[14], [38]. Additional power calculations suggested that integrated approaches to detect *cis* eQTL associations relevant to QTs in LCL expression data are likely to require much larger sample sizes than currently are thought necessary or available.

For many clinical phenotypes, comprehensive collections of relevant human tissues are not commonly available. Although previous studies have found a large number of eQTLs detected in LCLs with large effect sizes which could also be detected in primary tissues [21], [22], the question is whether these LCL expression profiles have any relevance to downstream clinical traits. When we focussed on expression probes nominally correlated with clinical QTs, we did not detect any large effect eQTLs for these probes. Moreover, several studies have highlighted cell type- or tissue specific genetic effects where others found that a substantial numbers of eQTLs are shared across tissues [12], [20]. Hence, the detection of tissue-specific eQTLs with smaller effect sizes is likely to be more important for QTs.

Although the proportion of expression variance explained by the *cis* eQTL SNPs in our analyses appeared considerable, effect sizes derived from small datasets are likely to be subject to the ‘winner's curse’ [40]. In the overlap analysis of three HapMap datasets, the proportion of expression variance explained by each of 58 *cis* eQTL SNPs ranged from 17–46%, yet only two of these signals were replicated in the Twin V2 analysis. Whilst we do not currently know the spectrum of effect sizes for *cis* eQTL SNPs, and assume that some of these will be of considerable magnitude, the effect sizes of *trans* eQTLs are likely to be much smaller and similar to those observed in complex traits. Moreover, the additional multiple testing burden inherent to *trans* vs. *cis* analysis further increases the likely sample sizes required.

It is perhaps not surprising that only one suggestive eQTL-QT association among 44 QTs was observed in our study. In addition to the size of the datasets employed for eQTL detection, we were limited by the size of the twin sample with available LCLs (N = 299) to detect expression probe-QT correlations. Nevertheless, our results showed that the expression probe sets filtered at each stage of analysis had progressively increasing heritability levels, suggesting an enrichment of genetically relevant signals. Further limitations arose because only 76% of the QT-correlated probes were annotated across different expression platforms. Testing SNPs tagging the eQTL SNPs for association with the relevant QT in the twin cohort, we were able to follow up only 70–75% of the eQTL SNPs due to the coverage of the available genotyping data. This strategy will result in a loss of power compared with testing the eQTL SNP itself, but has been minimised by focussing on highly correlated tags (r^2^>0.8, at least 80% power of eQTL SNP). An alternative approach to increase power would be to perform imputation in the twins, although current methodology [41] is not well calibrated for related individuals. In addition, we were able to test only 56% of our results for replication in large cohorts with GWA data and we could only test the most promising association between rs1051140 and phosphate levels, based on a *cis* eQTL signal, in an independent dataset of 1,756 individuals. Given the sample sizes required for the GWA detection of SNPs with QTs, the size of this replication cohort may have been insufficient. Although some of these problems may be resolved with the advent of large-scale genotyping and gene expression profiling in large epidemiological biobanks, they are nevertheless real issues inherent to currently available datasets.

Because the physiology of specific cell types changes with disease/QT, or the phenotype is manifested in specific tissues exclusively, it appears logical that correlations between QT and gene expression should be detectable in tissues relevant to the QT in question. Although the number of nominally significant correlations between QT and expression probes was more than expected by chance in our study, it was lower than in previous studies using gene expression profiling on liver or adipose tissue or blood [12], [14] suggesting that the effect sizes in LCLs might be reduced compared to expression profiling *in vivo* tissues. Indeed, in a recent study, 72% of the expression traits identified from adipose tissue were significantly correlated with BMI whereas in blood this was only 9%. More than 10% of the BMI variation was explained by 16% of these expression traits whereas none of the correlated expression traits in blood reached this level of correlation [12].

The extent to which eQTLs will be shared across tissues and cell types is still unknown. We failed to confirm previously published eQTL-QT associations derived from fresh lymphocytes, adipose and liver tissue in the LCLs. The low percentage of overlap of probe detection (66%) in LCLs suggests that some transcripts are only expressed in specific tissues or have a better annotation on other platforms. Indeed, in an eQTL analysis of three cell types (LCLs, fibroblasts and T-cells), Dimas *et al*. (2009) detected cell type-specific genetic effects, with 69%–80% of genetic variants acting in a cell type-specific manner [20]. Emilsson *et al.* (2008) showed little difference in the number of *cis* eQTLs mapped in adipose tissue and blood with approximately 50% overlap confirming that there is both common and tissue-specific genetic control [12]. Highly significant *cis* eQTLs (unrelated to disease/QT) have been found in LCLs [6]–[8], [11] and some of these eQTLs findings were replicated in lymphocytes [6], [13]. Nica *et al*. (2010) and Nicolae *et al*. (2010) observed a significant overrepresentation of *cis* eQTLs in the LCLs of the HapMap individuals among GWAS SNPs for immunity related traits as compared to random SNPs [42], [43]. Taken together, we speculate that the utility of LCLs might be more powerful for traits with a more direct immunological relevance.

The reproducibility of gene expression measurements has been questioned, and lack of concordance attributed to the small sample sizes and technical variability although this might also be a reflection of the different annotation or statistical methods used [24]–[26]. In our integrative approach, we found a few potential QT-eQTL signals (a *cis* eQTL for phosphate and a *trans* eQTL for height) which emerged only after three individually underpowered eQTL HapMap datasets of HapMap founders were combined at a nominal significance level. Notably, we also found suggestive evidence for these eQTL associations in the 161 twins for which we had genotypic and gene expression data available. This highlights the fact that although the use of multiple gene expression HapMap datasets can reduce the number of false positive results caused by platform-specific technical artefacts (technical replication), it cannot resolve low power to detect eQTL signals due to small sample size (requiring biological replication) and is hampered by the reduced number of the QT-correlated probes (76%) that were annotated across platforms.

A few studies have adopted a different approach to detect QT-related expression traits with small effect sizes by constructing of co-expression networks. Specifically, they identified modules of co-expressed probes and investigated the average correlation between the expression probes in the module and obesity-related clinical traits [12], [14]. This is a potentially attractive and more powerful approach, given that in theory it better reflects the complex nature of gene expression networks, in which many expression traits will not necessarily be causative of but mostly reactive to disease, and in which each individual expression trait is likely only to have a small effect [44]. Networks built on correlations between expression traits, however, are likely to be noisy, as ultimately any biases (experimental or otherwise) acting on expression levels may cause artificial results if correlated with the clinical trait in question. Although promising, it is at present unclear to what extent the limitations that have hindered single expression trait analyses impact on network or other types of multivariate analyses of expression traits.

Although integrating genotype and gene expression with multiple QTs in large datasets has the potential to improve our understanding of common traits, our study found that approaches using LCLs with currently available sample sizes are underpowered. To detect associations between eQTLs and clinical phenotypes, larger sample sizes are required and – if available - datasets profiled on a tissue relevant to the clinical trait in question should also be used. Such datasets will become available in the near future, enabling a fuller exploration of the use of integrated ‘omics’ analyses in uncovering genetic origins of complex traits.

## Methods

### Study participants and QTs (Stage I & Stage III)

Study subjects comprised 6,533 female twins (age-range 19–81 years) from the St Thomas' UK Adult Twin registry (www.twinsuk.ac.uk). Twins were recruited from the general population through national media campaigns in the UK and shown to be comparable to age-matched population singletons in terms of disease-related and lifestyle characteristics [45]. The study was approved by St Thomas' Hospital Research Ethics Committee and all twins provided informed written consent.

Zygosity was determined with a standardized questionnaire and confirmed through genotyping [46]. The study was approved by St Thomas' Hospital Research Ethics Committee and all twins provided informed written consent. Administered questionnaires provided information for an extensive range of demographic variables and medical history. We selected 44 QTs including blood pressure (systolic and diastolic blood pressure (first and second measurements), sodium), BMD (lumbar spine, total hip, femoral neck, and total forearm), bone (alkalinephosphatase, calcium, vitamin D, phosphate and crp), and liver function markers (albumin, bilirubin, total protein concentration and gamma glutamyl transpeptidase activity), fat-related (triglycerides, weight, BMI, total fat mass(g), total lean mass(g), central fat mass (g), total fat mass (%), central fat mass (%), hip and waist circumference, leptin, and adiponectin) and glycemic traits (fasting insulin and glucose concentrations), electrolytes (bicarbonate, urea, albumin corrected calcium, chloride, magnesium and potassium) and lipids (cholesterol, HDL and LDL) and other (creatinine, height and telomere length) (Text S1). The total sample of female 6,533 twins (1,428 monozygotic (MZ) and 1,829 dizygotic (DZ) pairs, 12 singletons, one trio and a quadruple) was used for QT heritability calculations. For the 44 QTs, phenotypic data was available between 37%–99% of the twins. LCLs were available for 299 (101 MZ and 43 DZ pairs and 11 singletons) of the 6,533 twins of which had between 43%–99% phenotypic data available (Stage I). For the confirmation of QT-eQTL SNP associations, genotypic data (Human Hap 300 k Duo chip, (Illumina, San Diego, USA)) was available for 2,905 of the 6,533 female twins. For 161 of the 2,905 female twins (86 families), LCL gene expression and genotypic data was available.

### Gene expression datasets

In Stage II, three datasets consisting of 60 CEU HapMap founders were used: one publicly available dataset and two generated for this study. The 299 twins were profiled using the Illumina WG-6 Expression BeadChip V2; the 60 HapMap individuals on the Illumina WG-6 Expression BeadChip V1 and V2 (Table S3) (referred to as WTCHG V1 and V2 respectively). In addition, we downloaded a publicly available gene expression dataset [11] from the Gene Expression Omnibus website (GSE6536) corresponding to the same HapMap individuals and profiled on the Illumina WG-6 Expression Bead Chip V1. The Illumina Human WG-6 V1 Sentrix BeadArray contains 47,296 probes representing 24,385 RefSeq annotated transcripts. The Illumina Human-6 V2 Sentrix BeadArray contains 48,702 unique probes representing 28,567 RefSeq annotated transcripts. LCLs for the 299 twins were generated by the European Collection of Cell Cultures, and cell pellets transported to the WTCHG. LCLs from the 60 HapMap CEU individuals were purchased from Coriell and cultured at WTCHG. The resultant data were parsed with the software package BeadStudio (Illumina software) to produce raw intensity values for all probes. Signal was checked for quality using hybridisation and labelling controls internal to each array and subtracted for background within the statistical scripting environment, R v2.9.1 (http://cran.r-project.org/). Signal was transformed and normalised using the variance stabilization algorithm (vsn) and quantile normalization as implemented in the vsn2 Bioconductor (http://www.bioconductor.org/) package [47]. Transformed and normalised signal distributions for each sample were investigated with unsupervised analysis and three outliers NA11829, NA11839, NA12056 were removed from WTCHG V1 and V2. We selected probes with an Illumina score of >0.95 in at least six individuals (10%) and had no SNPs with MAF>5% in their sequence, corresponding to 19,573 (twins), 20,200 (WTCHG V2) and 17,484 (WTCHG V1); 18,838 probes overlapped between the twins and WTCHG V2. As detection scores were not provided for the Stranger V1 dataset, the full dataset was used [11]. Expression probes were sequence matched to NCBI Build 36.1 (hg18) using the blastn algorithm to obtain a physical position from which Ensembl transcript identifiers were extracted and matched between Illumina's expression profiling arrays. (Text S1, Table S3 and S4).

### Statistical analysis

#### Correlations between QTs and gene expression probes (Stage I)

For QTs measured at multiple time points, the timepoint closest to extraction of the lymphocytes was used. Outlying values for each QT were identified and removed on basis of the distribution of the larger twin cohort. Skewed distributions of QTs were logarithmically or square root transformed to normalise distributions before analyses. To investigate the correlation structure of the QTs, PCA was performed using the NIPALS algorithm and Pearson correlations among the 44 QTs were calculated.

Phenotype correlations between 19,573 normalised gene expression levels and 44 QTs were computed using mixed models (lmer) implemented in the R package lme4. For logistical reasons beyond our control, twin pairs were always assessed and measured together. Mixed models were adjusted for correlation of measurements due to relatedness of the twins.

Possible confounders were identified by comparing the log likelihood of the mixed model with and without the possible confounder for each phenotype. In the mixed model, we included age, number of flask days, RNA Integrity Number as fixed effects and twin relationship as random effect.

#### eQTL analyses in the HapMap and Twin datasets (Stage II)

Nominally significant correlated expression probes (P<10^−3^) were tested for association in *cis* or *trans* with 2.2 million autosomal SNPs (r^2^<1.0; MAF >0.05, HapMap release 22, NCBI build 36) in three datasets of CEU HapMap founders or with 296,308 SNPs (MAF>0.05) in 86 unrelated twins that were profiled and genotyped from Stage I. Probes selected in Stage I (939 probes) that were not mapped to an autosomal genomic location in build 36 or mapped to multiple locations resulting in 718 probes used in the WTCHG V2 analysis or Twin V2 analysis. We were able to annotate 546 of the 718 V2 probes to 1,050 V1 probes and this set was analysed in the overlap analysis consisting of three HapMap datasets. For each of the selected expression probes and for each SNP, an one degree of freedom test (Wald test) was fitted using PLINK [48]. The genotypes were coded as 0, 1 and 2 and these allele counts were tested for an additive genotypic effect with the normalised gene expression values in each of the three HapMap datasets separately or Twin V2 dataset. Nominal significance thresholds of p<1*10^−3^ in all three HapMap datasets or p<5*10^−7^ (a commonly used GWA threshold previously employed by the Wellcome Trust Case Control Consortium [3]) in the WTCHG V2 or Twin V2 dataset were applied. Fixed-effects meta-analyses on WTCHG V2 and Twin V2 for 229 eQTL associations were performed using estimates of the allelic effect size and standard error [31]. eQTL associations from the overlap analyses and meta-analyses were checked for different directional genotypic effects. Associations in *cis* were defined as a SNP-probe association where the distance from probe genomic midpoint to SNP genomic location was less than or equal to 1 Mb [11]. All other associations were defined as associations in *trans* [11]. Given the redundancy of the V1 probes compared to the V2 probes and the correlation between expression probes, we used Illumina V2 probe identifiers as an annotation reference.

#### SNP-QT associations in the twin cohort (Stage III)

For the TwinsUK cohort, 2,167 samples were successfully genotyped with the Illumina HumanHap 300 k Duo Beadchip (Illumina, San Diego, USA). For the second individual of a MZ pair, genotypes were copied from the other genotyped MZ individual of the pair. After applying strict quality control filters we retained 296,308 autosomal SNPs with a MAF>0.05 and 2,905 female twins (Text S1). From the eQTL analysis in Stage II, we selected 1,989 eQTL SNPs from the overlap analysis and 1,932 eQTL SNPs from the WTCHG V2 analysis. Pairwise r^2^ values and MAF from the International HapMap Project [30] were used to assess which SNPs were in LD (r^2^>0.8) with the eQTL SNPs and to compare MAF distributions. From the SNPs in LD with the eQTL SNPs, 535 SNPs for the overlap analysis and 451 SNPs for the WTCHG V2 analysis were present on the Illumina HumanHap 300 k Duo Beadchip, successfully genotyped in the twin cohort and had passed QC. These SNP sets, which tagged (r^2^>0.8) 1,482/1,989 of the eQTL SNPs from the overlap analysis and 1,357/1,932 from the WTCHG V2 analysis, were tested for association with 37 QTs and 41 QTs, respectively. In addition, 242 SNPs from the Twin V2 and 101 SNPs from the meta-analysis were tested for association with 38 QTs and 36 QTs, respectively. Genotyped eQTL SNPs or their proxies were tested for association with the correlating QT in the twin cohort using a linear mixed model adjusted for family relationship and zygosity. We tested the additive genotypic effect of the SNP, coded as 0, 1, and 2 with the transformed QT using the lmer function in the lme4 R package and assessed significance by applying a FDR [33]. Adjustment for age did not modify the association between the SNPs and the clinical phenotypes (except for telomere length, data not shown). SNP-QT associations (p<0.01) were checked for outlying values in gene expression distributions. For the remaining 16 SNP-QT associations with p<0.01, eQTL associations were tested in the full set of 86 twin families (161 individuals), using a linear mixed model adjusted for family relationship and zygosity.

#### Follow-up of SNP-QT associations in independent cohorts (Stage IV)

In order to confirm the nine SNP-QT associations in independent cohorts, we obtained results of relevant meta-analyses conducted by five different consortia. To confirm the height association, three SNPs (rs1482455, rs9342097, rs9353441) were tested in 5,828 European females from three GWA studies: the British 1958 Birth Cohort, the Rotterdam Study and EPIC Norfolk study. Meta-analysis statistics were obtained using a weighted z-statistics [49]. For the three crp associations, five SNPs were examined for association in a subcohort (Genmets) of the Health2000 population cohort consisting of 1,097 European women with metabolic syndrome and matched healthy controls using an additive model adjusted for case-control status and age [34]. SNP (rs2266917) was tested for association with triglyceride levels in the ENGAGE cohort datasets comprising 16,129 individuals from 14 GWA studies (excluding TwinsUK individuals) using a fixed-effects meta-analysis with reciprocal weighting [35]. For the associations with diastolic blood pressure, three SNPs (rs200969, rs149900 and rs12150466) were examined in 26,644 women of the Global BPgen consortium blood comprising 17 GWAS studies using inverse variance weighting [36]. In the population-based KORA study comprising two follow-up populations of 1,756 (F3:829, F4:927) women of European descent, we examined two SNPs, rs10511409 and rs7647266, for association with phosphate levels using an inverse variance meta-analysis [37].

### Gene set enrichment, heritability estimates and power calculations

We used DAVID to conduct GO term enrichment analysis [32]. A Fisher Exact test was used to assess significance and a FDR was used to adjust for multiple testing [33]. Heritabilities were calculated using a structural equation modelling framework in Mx [50]. Sample sizes needed to detect eQTLs with effects varying between 0.01 and 0.4 of QTL variance explained, assuming a significance threshold α of 5*10^−7^ or 1.0*10^−3^, and 50% or 80% power, were conducted using the Genetic Power Calculator [39].

## Supporting Information

Text S1 Supplemental methods.
(DOC)

Figure S1 Correlation structure among the 44 QTs. Heatmap displays correlation structure among the 44 QTs using pearson correlations and hierarchical clustering.
(TIF)

Figure S2 Minor allele frequencies of untagged eQTL SNPs. A) Minor allele frequencies of untagged SNPs from the overlap eQTL analysis. B) Minor allele frequencies of untagged SNPs from the WTCHG V2 eQTL analysis.
(TIF)

Table S1 Number of expression probes correlated with more than two QTs at p<10^−3^.
(XLS)

Table S2 The correlation between expression probes and QTs with and without adjustments for smoking for expression probes that are correlated with smoking.
(XLS)

Table S3 Expression profiling of 299 female twins and three datasets of 60 HapMap founders.
(XLS)

Table S4 Expression probe mapping between different Illumina platforms based on Ensembl transcript identifiers using BLAT and BLAST (NCBI build 36).
(XLS)

Table S5 Number of eQTL SNPs tagged by SNPs on GWA array (r^2^>0.8).
(XLS)

Table S6 Correlations between gene expression probes and QTs from the literature tested in the 299 twins.
(XLS)
