# Supplementary material for: The Use of Genome-Wide eQTL Associations in Lymphoblastoid Cell Lines to Identify Novel Genetic Pathways Involved in Complex Traits
Source: PLoS One. 2011 Jul 15;6(7):e22070. doi: 10.1371/journal.pone.0022070 (PMC3137612; doi:10.1371/journal.pone.0022070)
Supplement: Text S1 — Supplemental methods. (DOC) [file pone.0022070.s001.doc]

**Text S1**

**Supplemental methods**

***Clinical phenotypes and biochemical assays***

Subjects were asked if they were currently smoking cigarettes daily. Brachial BP was measured with an automated cuff sphygmomanometer (OMRON HEM713C) with the subject in the seated position under standardized conditions. The average of 2 BP readings was recorded [1]. Height was measured using a stadiometer, and weight was recorded while the subjects were wearing light street clothing and no shoes. BMI was calculated by dividing weight in kilograms by the square of height in metres. Hip and waist circumference was determined in centimetres with tape measure. Waist circumference was measured at the level midway between the lower rib margin and the iliac crest. Body composition (total fat mass, central fat mass and total lean mass) were measured using dual energy x-ray absorptiometry (QDR 2000W, Hologic, Bedford, MA) and calculated using standard software (version 710). Central abdominal fat was measured by a blinded investigator and was defined as the abdominal region delineated by the second lumbar to the fourth lumbar vertebrae, and laterally to the inner aspects of the ribs [2]. BMD had been measured at lumbar spine (L1-L4), total hip, femoral neck, and total forearm using dual energy x-ray absorptiometry (QDR 2000W, Hologic, Bedford, MA)[3]. Total cholesterol, LDL, HDL, and triglycerides were determined by a colorimetric enzymatic method [4]. Fasting serum insulin and glucose concentrations were measured by using methods describes previously [5]. Fasting serum insulin concentrations were assayed by using a chemiluminescent Immulite kit (Diagnostics Products Corp, Los Angeles, CA). Glucose was measured on an Ektachem 700 multichannel analyser using an enzymatic colorimetric slide assay (Johnson and Johnson Clinical Diagnostic Systems, Amersham, UK). Fasting morning serum total adiponectin levels were measured with a two-site DELFIA assay using antibodies and standards from R&D Systems (Minneapolis, MN). Serum leptin concentrations were measured after the subjects had fasted overnight by using a radioimmunoassay (Linco Research, St Louis, MO). 25-Hydroxyvitamin D concentrations were measured by using a radioimmunoassay kit (DiaSorin Inc, Stillwater, MN) [6]. Serum CRP concentrations were measured by using an enzyme-linked immunosorbent assay. Assays for serum calcium, magnesium, phosphate and creatinine were performed on a 950 Vitros analyser (Ortho-Clinical Diagnostics; Johnson and Johnson, Rochester, NY, U.S.A.). Urinary sodium and potassium were measured on a Corning 480 Flame photometer. Urea, bicarbonate and chloride were measured with autoanalyser. Serum calcium concentrations were corrected for albumin using the following equation: CCa(Alb) =Ca+0·016(41-Alb), where Ca, Alb and CCa(Alb) are serum total calcium (mmol/l), albumin (g/l) and serum calcium corrected for albumin (mmol/l), respectively [7,8]. The mean leukocyte telomere length was assessed using the terminal restriction fragment length which was measured using the Southern blot method in duplicates as described previously [9]. Fasting blood samples were collected from all twins for biochemical assays of the liver function proteins. All liver function assays were performed on a Synchron LX20 automated multi channel analyzer (Beckman Coulter, Fulleton, CA). Serum albumin concentration was measured by means of a bichromatic digital endpoint methodology using bromcresol purple reagent. Gamma glutamyl transpeptidase activity was measured by an enzymatic rate method. Bilirubin was measured by a timed endpoint Diazo method. Total protein concentration was measured by means of a rate biuret method. Alkaline phosphatase activity was measured by a kinetic rate method using a 2-amino-2-methyl-1-propanol buffer [10].

***RNA preparation and preprocessing***

Frozen stocks from previously immortalised cell lines were cultured in 10% fetal calf serum in a 5% CO_2_ incubator. Cells were harvested at approximately 60% confluency by centrifugation to a cell pellet of on average 10^8^ cells. RNAlater® was added to the cell pellet and stored frozen. The cell solution was thawed, centrifuged to a cell pellet and RNAlater® removed. The cell pellet was resuspended in Qiagen RLT buffer and passed through a 20 gauge needle to disrupt cellular integrity and the QIAGEN RNeasy mini protocol was used to extract total RNA. RNA was quantified using a NanoDrop and checked for quality using the Agilent Bioanalyser. 500 ng of total RNA was labelled using the Ambion TotalPrep amplification kit. Individual samples were randomised before RNA extraction, amplication, and hybridisation to Illumina Human WG-6 Sentrix BeadArray arrays. Arrays were hybridised with labelled cRNA material and scanned according to manufacturer's instructions.

We used publicly available gene expression levels from Stranger et al. (2007) (Illumina v1 Beadchips) as a third HapMap eQTL discovery set. Briefly, for the 210 HapMap founder lymphoblastoid cell lines, Stranger et al. (2007), ran two in vitro transcription reactions and ran each reaction on two plates, giving a total of four runs per individual. Using a quantile normalization method raw data was normalized across replicates of a single individual and then individuals with each population were median centralized [11].

The detection score of a probe is a quality score between 0 and 1 representing the signal-noise ratio. We calculated interindividual correlations for probes with identical sequences between the two chips using different detection score thresholds. Interindividual correlations for probes that are detected in more than 10% of the individuals showed mean spearman correlation of 0.96 (range 0.90-0.98). In our analyses, we included probes that are detected in at least 10% of the individuals with a detection score larger than 0.95. Detection scores were not available for the published dataset and therefore we used all 47,293 probes.

***Smoking as confounder***

Given the potential confounding effect of smoking on the association between QTs and expression values, we investigated the effect of smoking on gene expression levels. We classified the 299 twins in 128 never smokers, 81 former smokers and 50 current smokers. We tested the 956 expression probes for association with smoking using the same linear mixed model described above. At a nominal significance value of p <10^-3^, 12 expression probes were correlated with current smoking. We next determined the correlation between these probes and the QTs with and without adjustments for smoking to investigate whether a QT correlation was mediated by smoking. For eight out of the 12 probes, expression probe-QT correlations (triglycerides, diastolic blood pressure (three probes), height, crp, albumin (calcium corrected) and calcium) were observed when adjusted for smoking, but disappeared without adjustments for smoking in the model (Supplemental Table 2). To account for the fact that correlations might disappear due to small fluctuations in the pvalue close to our nominal significance threshold, we calculated the direct correlations between these QTs and smoking in the mixed model; diastolic blood pressure was the only QT that showed a correlation with smoking (p=5.5*10^-5^). In summary, ILMN_16141 targeting *ZNF548* correlated with diastolic blood pressure was the only expression probe-QT association mediated by smoking.

***Probe annotation***

To identify matched probes between Illumina’s expression profiling arrays, expression probes were sequence matched to NCBI Build 36.1 (hg18) using the blastn algorithm to obtain a physical position from which Ensembl transcript identifiers were extracted and matched (Supplemental Table 3 and 4). This approach was necessary for mapping within the Illumina platform versions as these share <1% (n=366) of probes with a common sequence. Unfortunately, none of these mappings are one to one: not every expression probe on the arrays has been annotated, some probes are annotated to multiple genomic locations, multiple Ensembl transcript identifiers or multiple probes on the other array. Probes that showed one mismatch or more were aligned to Ensembl transcripts or EMBL ESTs using BLAST (1), and genomic locations were then established by re-mapping the target transcript to genome (NCBI build 36) either by extracting annotation data from UCSC MySQL tables or by BLAST against genomic sequence. Probes overlapping at least 10 bases of repeat sequence, established by using RepeatMasker (2) on the transcript sequence, were discarded. Probes that have SNPs in their sequences or that had no match to the human genome build 36 were removed from the analysis. We could extract Ensembl transcripts identifiers for a total of 21,855 V2 and 25,492 V1 expression probes respectively. Of these ~20,000 expression probes had a matching probe in the other version corresponding to 27,853 Ensembl transcripts (Supplemental Table 4).

***Genotyping and quality control***

For the TwinsUK cohort, 2,820 Twins UK samples were genotyped for 318,232 SNPs from the Human Hap 300 Duo chip. Quality control on the genotyped subjects was performed applying quality control filters as described previously [12]. A further 35 individuals were removed. These individuals were: outliers, unrelated but with high kinship coefficients, related but with low kinship coefficients or failed gender check.

In addition, we applied quality control filters from the Welcome Trust Case Control Consortium [13] in two random groups of unrelated female twins. We removed 13 individuals with missingness of SNPs > 3%. Ethnicity has been examined on a pruned dataset (r2 >0.5) with principal components analysis implemented in Goldsurfer2 and compared with self-reported ethnicity from TwinsUK [14]. We excluded 17 individuals with non-Caucasian ancestry. SNPs were removed when MAF>5% and missingness > 0.05 or MAF<1% and missingness >0.01 or Hardy Weinberg equilibrium exact p value < 5.7*10^-7^. We retained 2,167 twins comprising one individual for each monozygotic twin pair and two individuals for each dizygotic twin pair. For the second individual of a MZ pair, genotypes were copied from the other genotyped MZ individual of the pair. Finally, we included 2,905 individuals and 296,308 autosomal SNPs in our analysis.

References

1. Snieder H, Hayward CS, Perks U, Kelly RP, Kelly PJ, et al. (2000) Heritability of central systolic pressure augmentation: a twin study. Hypertension 35: 574-579.

2. Samaras K, Spector TD, Nguyen TV, Baan K, Campbell LV, et al. (1997) Independent genetic factors determine the amount and distribution of fat in women after the menopause. J Clin Endocrinol Metab 82: 781-785.

3. Hunter DJ, de Lange M, Andrew T, Snieder H, MacGregor AJ, et al. (2001) Genetic variation in bone mineral density and calcaneal ultrasound: a study of the influence of menopause using female twins. Osteoporos Int 12: 406-411.

4. Middelberg RP, Spector TD, Swaminathan R, Snieder H (2002) Genetic and environmental influences on lipids, lipoproteins, and apolipoproteins: effects of menopause. Arterioscler Thromb Vasc Biol 22: 1142-1147.

5. de Lange M, Snieder H, Ariens RA, Andrew T, Grant PJ, et al. (2003) The relation between insulin resistance and hemostasis: pleiotropic genes and common environment. Twin Res 6: 152-161.

6. Jenkins AB, Samaras K, Gordon MA, Snieder H, Spector T, et al. (2001) Lack of heritability of circulating leptin concentration in humans after adjustment for body size and adiposity using a physiological approach. Int J Obes Relat Metab Disord 25: 1625-1632.

7. Hunter D, de Lange M, Snieder H, MacGregor AJ, Swaminathan R, et al. (2001) Genetic contribution to bone metabolism, calcium excretion, and vitamin D and parathyroid hormone regulation. J Bone Miner Res 16: 371-378.

8. Hunter DJ, Lange M, Snieder H, MacGregor AJ, Swaminathan R, et al. (2002) Genetic contribution to renal function and electrolyte balance: a twin study. Clin Sci (Lond) 103: 259-265.

9. Benetos A, Okuda K, Lajemi M, Kimura M, Thomas F, et al. (2001) Telomere length as an indicator of biological aging: The gender effect and relation with pulse pressure and pulse wave velocity. Hypertension 37: 381-385.

10. Rahmioglu N, Andrew T, Cherkas L, Surdulescu G, Swaminathan R, et al. (2009) Epidemiology and genetic epidemiology of the liver function test proteins. PLoS ONE 4: e4435.

11. Stranger BE, Forrest MS, Dunning M, Ingle CE, Beazley C, et al. (2007) Relative impact of nucleotide and copy number variation on gene expression phenotypes. Science 315: 848-853.

12. Richards JB, Rivadeneira F, Inouye M, Pastinen TM, Soranzo N, et al. (2008) Bone mineral density, osteoporosis, and osteoporotic fractures: a genome-wide association study. Lancet 371: 1505-1512.

13. The Wellcome Trust Case Control Consortium (2007) Genome-wide association study of 14,000 cases of seven common diseases and 3,000 shared controls. Nature 447: 661-678.

14. Pettersson F, Morris AP, Barnes MR, Cardon LR (2008) Goldsurfer2 (Gs2): a comprehensive tool for the analysis and visualization of genome wide association studies. BMC Bioinformatics 9: 138.

15. Emilsson V, Thorleifsson G, Zhang B, Leonardson AS, Zink F, et al. (2008) Genetics of gene expression and its effect on disease. Nature 452: 423-428.

16. Kathiresan S, Willer CJ, Peloso GM, Demissie S, Musunuru K, et al. (2009) Common variants at 30 loci contribute to polygenic dyslipidemia. Nat Genet 41: 56-65.

17. Goring HH, Curran JE, Johnson MP, Dyer TD, Charlesworth J, et al. (2007) Discovery of expression QTLs using large-scale transcriptional profiling in human lymphocytes. Nat Genet 39: 1208-1216.

References [15–17] are in Supplemental Table 6.
