# Supplementary figures and images for: The Use of Genome-Wide eQTL Associations in Lymphoblastoid Cell Lines to Identify Novel Genetic Pathways Involved in Complex Traits
Source: PLoS One. 2011 Jul 15;6(7):e22070. doi: 10.1371/journal.pone.0022070 (PMC3137612; doi:10.1371/journal.pone.0022070)

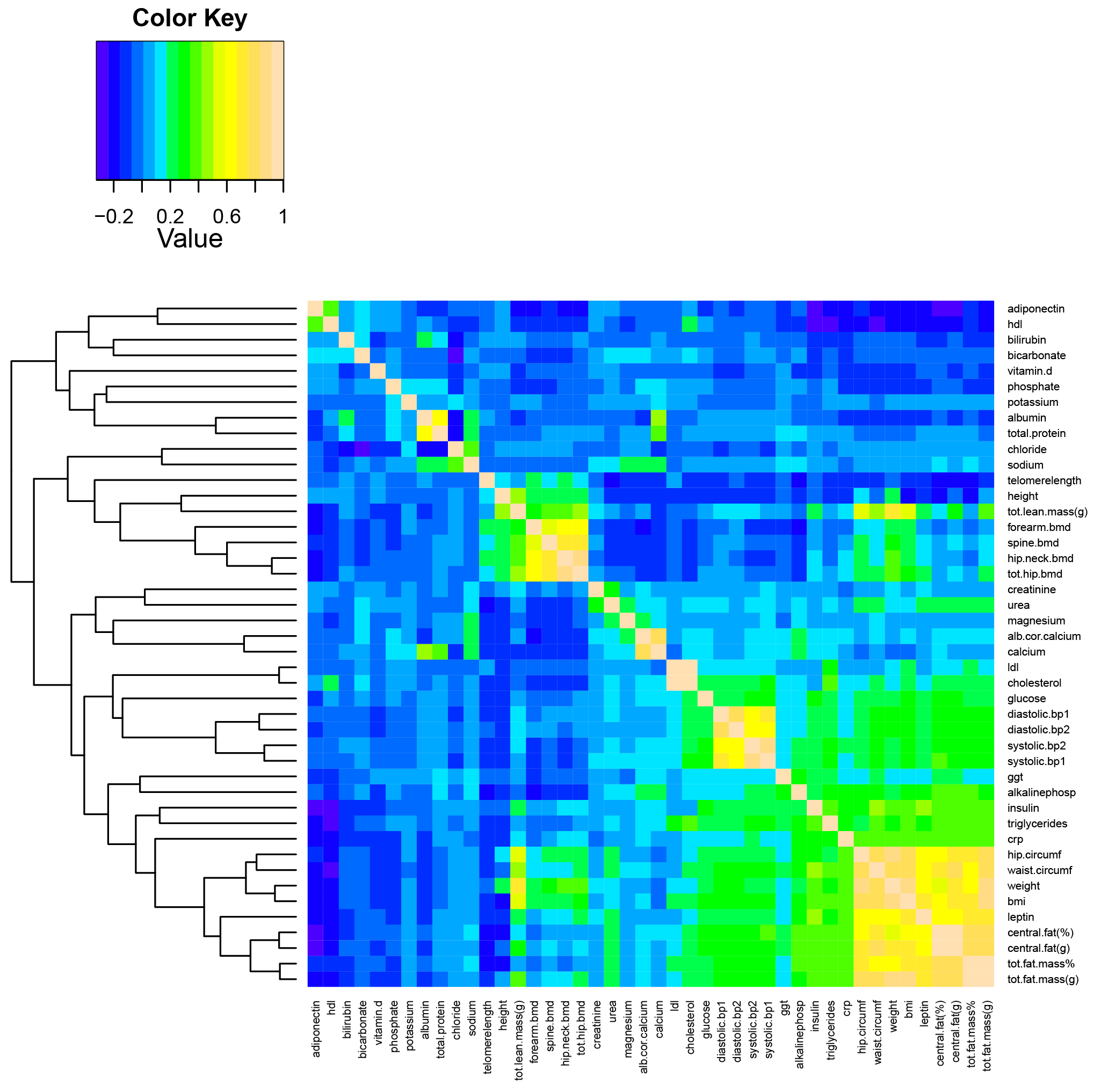

Supplement: Figure S1 — Correlation structure among the 44 QTs. Heatmap displays correlation structure among the 44 QTs using pearson correlations and hierarchical clustering. (TIF) [file pone.0022070.s002.tif]

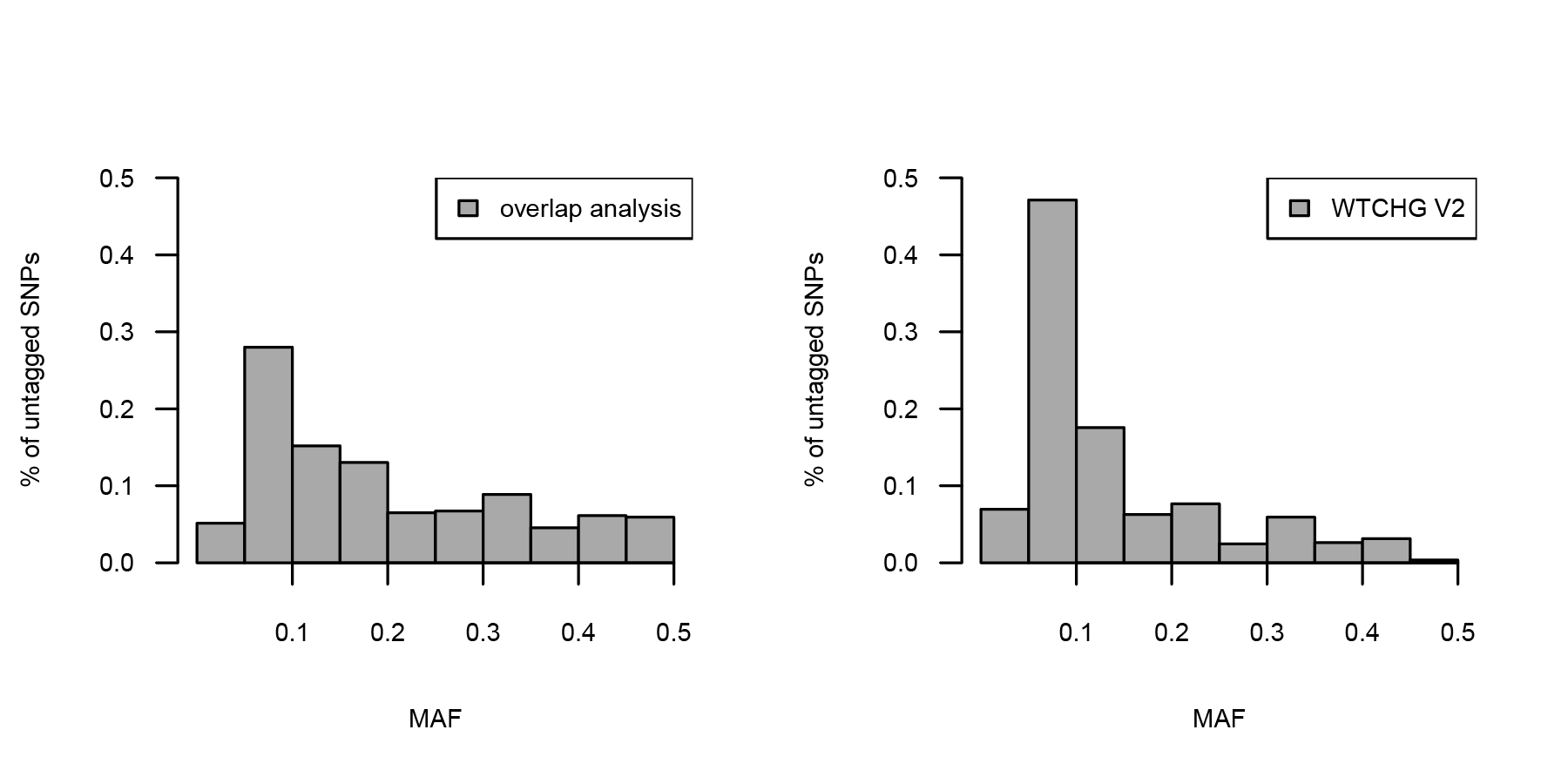

Supplement: Figure S2 — Minor allele frequencies of untagged eQTL SNPs. A) Minor allele frequencies of untagged SNPs from the overlap eQTL analysis. B) Minor allele frequencies of untagged SNPs from the WTCHG V2 eQTL analysis. (TIF) [file pone.0022070.s003.tif]
